# A Comparative Analysis of Climate-Risk and Extreme Event-Related Impacts on Well-Being and Health: Policy Implications

**DOI:** 10.3390/ijerph15020331

**Published:** 2018-02-13

**Authors:** Walter Leal Filho, Abul Quasem Al-Amin, Gustavo J. Nagy, Ulisses M. Azeiteiro, Laura Wiesböck, Desalegn Y. Ayal, Edward A. Morgan, Paschal Mugabe, Marilyn Aparicio-Effen, Hubert Fudjumdjum, Charbel Jose Chiappetta Jabbour

**Affiliations:** 1 School of Science and the Environment, Manchester Metropolitan University, Chester Street, Manchester M1 5GD, UK; walter.leal2@haw-hamburg.de; 2 Institute of Energy Policy and Research (IEPRe), Universiti Tenaga Nasional (UNITEN), Kajang 43000, Malaysia; 3 Department of Urban Studies & Planning, Massachusetts Institute of Technology, Boston, MA 77 Massachusetts Avenue, Cambridge, MA 02139-4307, USA; 4 Instituto de Ecología y Ciencias Ambientales (IECA), Facultad de Ciencias, Universidad de la República, Montevideo 11400, Uruguay; gnagy@fcien.edu.uy; 5 Department of Biology & CESAM Centre for Environmental and Marine Studies, University of Aveiro, 3810-193 Aveiro, Portugal; ulisses@ua.pt; 6 Department of Sociology, University of Vienna, Rooseveltplatz 2, 1090 Vienna, Austria; laura.wiesboeck@univie.ac.at; 7 Center for Food Security Studies (CFSS), College of Development Studies, Addis Ababa University, Addis Ababa 150129, Ethiopia; desalula@gmail.com; 8 Cities Research Institute, Griffith University, Nathan Campus, 170 Kessels Road, Nathan, QLD 4111, Australia; ed.morgan@griffith.edu.au; 9 Research and Transfer Center,“Sustainability and Climate Change Management”, Faculty of Life Sciences, Hamburg University of Applied Sciences, Ulmenliet 20, 21033 Hamburg, Germany; paschalmugabe@hotmail.com (P.M.); fudjumh@yahoo.fr (H.F.); 10 Facultad de Medicina—Instituto Boliviano de Biología de Altura (IBBA), Universidad Mayor de San Andrés (UMSA), Unidad de Cambio Climático, Ambiente y Salud, Claudio Sanjinez S/N, Miraflores, La Paz, Bolivia; marilyneffen@gmail.com; 11 Montpellier Research in Management, Montpellier Business School, 34000 Montpellier, France, cjcjabbour@gmail.com

**Keywords:** climate-change adaptation, extreme events, health, socioeconomic issues, vulnerability, adaptive capacity, environmental risk, adaptation strategies

## Abstract

There are various climate risks that are caused or influenced by climate change. They are known to have a wide range of physical, economic, environmental and social impacts. Apart from damages to the physical environment, many climate risks (climate variability, extreme events and climate-related hazards) are associated with a variety of impacts on human well-being, health, and life-supporting systems. These vary from boosting the proliferation of vectors of diseases (e.g., mosquitos), to mental problems triggered by damage to properties and infrastructure. There is a great variety of literature about the strong links between climate change and health, while there is relatively less literature that specifically examines the health impacts of climate risks and extreme events. This paper is an attempt to address this knowledge gap, by compiling eight examples from a set of industrialised and developing countries, where such interactions are described. The policy implications of these phenomena and the lessons learned from the examples provided are summarised. Some suggestions as to how to avert the potential and real health impacts of climate risks are made, hence assisting efforts to adapt to a problem whose impacts affect millions of people around the world. All the examples studied show some degree of vulnerability to climate risks regardless of their socioeconomic status and need to increase resilience against extreme events.

## 1. Introduction

The impacts of climate change and extreme events are felt across the globe, to different degrees [1]. Some regions have already experienced costly impacts of extreme events in terms of loss of lives, economic damagethat includes damage to property, infrastructure, agriculture, coastal forests and tourism, and food insecurity and health impacts [2,3]. This is due to the observed changes in the frequency, intensity, and duration of most of these extreme events [4].

Climate-Change impacts on health require the articulation of transdisciplinary research. This entails the collaboration and coordination of researchers from different disciplinary backgrounds, intersectoral and interinstitutional, where a particular focus on the transdisciplinary study can play a fundamental role [5,6].

Climate change is known to be associated with the more frequent and intense occurrence of uneven extreme events. The increasing evidence of the links between extreme El Niño events and global warming suggest that the occurrence of such uneven extreme events could increase in the future due to climate change [7]. The risks associated with extreme events are expected to increase, as global mean temperature rises which, in turn, is likely to trigger disasters and vulnerability [8]. Even though they do not occur often, extreme events can be harmful to human health and can lead to damage to infrastructures, affect the economy, and even cause loss of life [9].

The primary effects of climate change on human health come from the direct impacts of physical systems on human well-being, such as heatwaves, floods and droughts. Indeed, heat stress directly caused by heatwaves may lead to heat-related illnesses such as hyperthermia, heat syncope, heat exhaustion, heat stroke, dehydration, and hypothermia. In addition, subjective elements such as perceived thermal discomfort may also lead to reduced work capacity and the lowering of performance. Moreover, when combined with existing illnesses such as cardiorespiratory diseases, this may pose serious health risks to the population, especially to vulnerable groups such as the elderly and young children. Managing the risk of extreme events under climate change is, therefore, crucial [1,2,8,10] considering its interlinkages with health and well-being. 

Preventing and reducing disaster risk in the context of the Sendai Framework for Disaster Risk Reduction 2015–2030 (SFDRR) implies understanding disaster risk, strengthening disaster risk governance (to manage disaster risk), investing in disaster reduction (for resilience), and enhancing disaster preparedness for effective response. SFDRR is the first global policy framework of the United Nations’ post-2015 agenda and it represents a step in the direction of global policy coherence with explicit reference to health, development and climate change [11]. Vulnerable communities and populations in developing countries are more affected by extreme events and climate change than industrialised countries, which can better cope with their impacts [8,12]. This state of affairs illustrates how mitigation and adaptation strategies are important to them.

There are various sectors of the population which have greater vulnerability to health impacts of extreme events; these include the elderly, children, and persons with existing health problems and disabilities [13]. Generally, the categories of extreme events with important health impacts include [14]:

(i) flooding related to hurricanes and coastal storms;
(ii) droughts through long dry spells, wildfires;
(iii) winter storms and severe thunderstorms.

The health impacts of these events are manifold and include:

(i) stress and pressures on mental health due to damage to properties;
(ii) outbreaks of mosquito-borne diseases, especially after floods;
(iii) malnutrition due to restrictions in the availability of crops, especially but not only due to crop failure.

These are already felt severely in areas such as the Pacific region, Africa, Asia and Latin America, where the limited capacity to address them make their impacts felt more strongly than elsewhere. The coupling of climate change and rapid urbanisation amplify the risks to human health. The demands for adequate and safe housing, clean water and sanitation, and access to health and food need to be combined with the need for protection from natural hazards [15]. 

Against this background, there is a need to mainstream the connections between climate change and health at all levels in order to better cope with increasing threats from extreme events [16]. However, there is a very limited understanding of how extreme events, as sources of vulnerability, are associated with the health and well-being of people. The recent Rockefeller Foundation–Lancet Commission report on Planetary Health, calls for an improved understanding of urban health and its interactions with climate change through a governance lens [17].

Despite the growing momentum at the international and regional levels, there is a need for more initiatives to be undertaken to examine the place-based interactions between climate change, urbanisation, climate variability, extreme events, and well-being and health. However, (i) such studies need to be performed on a large scale and over long periods of time; and (ii) they are very expensive. Therefore, the ambition of this self-funded research was more to describe the links between climate risks and health, by amassing the available literature on the one hand, and by collecting evidence from the participant examples on the other. This is on the rare occasions where such analysis has been performed, and the combination of countries, regions and cities used is unprecedented in the literature. The idea is to make readers more aware of such connections and illustrate which risks should be taken into account.

This article aimed to make a cross-comparison study which looked at a set of eight selected examples worldwide about the impacts of climate risks to human well-being, safety and health, where their occurrence, and impacts, were analysed.

The working hypothesis is: “All countries, regions and cities worldwide show some degree of vulnerability, are exposed to and are directly and/or indirectly impacted by climate-related hazards regardless of their socioeconomic status and readiness”. The eight highly diverse and even disparate surveyed examples are assumed to be representative of this statement. Some of the principal conclusions are as follows:

(i) Climate change is associated with extreme events and influences public health policies.
(ii) Extreme events are known to cause human well-being and health problems.
(iii) Vulnerable communities (e.g., the poor and those more exposed) are often affected, and sound mitigation and adaptation strategies may help them.
(iv) Developing countries are disproportionally affected by extreme events, but all countries are vulnerable.
(v) Resilience to extreme events and policies to support it are needed.

## 2. Materials and Methods

Based on the need for research which may better describe the links between climate risks and extreme events and health, a cross-comparison survey and analysis of eight examples was performed by compiling the most relevant works the authors were able to access. There can be no claim that the data represents the countries’ entire circumstances, but it does offer a profile of current trends which is what this paper tries to achieve. Unfortunately, there are few international studies that address the climate–health nexus in an industrialised and developing country context.

The methodology of the study consisted of three steps:

(i) Step 1: Identification of the sample and compilation of a list of climate risks and extreme events (CREE) relevant for human well-being, health and safety in the surveyed examples;
(ii) Step 2: Collection of evidence-based statistics; and
(iii) Step 3: A comparative analysis of climate-risks and extreme events, and health.

The methodological processes are addresses below.

### 2.1. Identification of the Sample and Compilation of a List of Climate Risks and Extreme Events (CREE)

**Figure 1:**
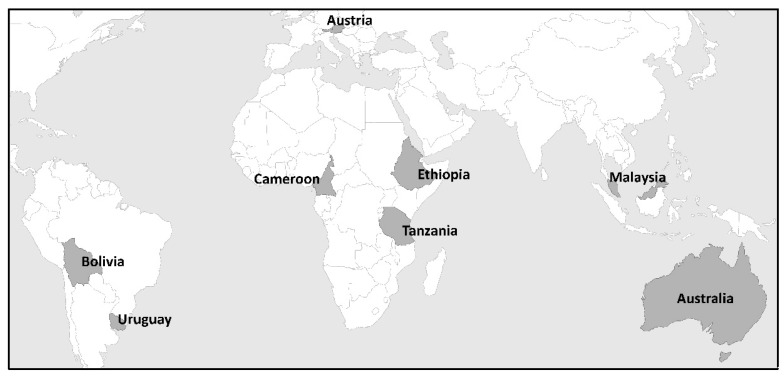
Study countries and regions.

This step identified the sample and compiled a list of CREE relevant to the places sampled. The sample consisted of eight highly different examples including four countries (i: Austria; ii: Ethiopia; iii: Malaysia; and iv: Uruguay), one large region (South East Queensland (SEQ) in Australia), and three cities (i: La Paz, Bolivia; ii: Douala, Cameroon; and iii: Dar-es-Salaam, Tanzania) (Figure 1). The rationale behind the choice of examples was the need to represent various regions and contexts based on the geographical location or origin of the scientists involved in the study and to consider the realities of industrialised, advanced transitional and developing nations.

National-level indicators are required for the use of international organisations, whilst local-level indicators are required by local-level governments [18]. However, due to the lack of appropriate comparative indicators for the studied cities, only the national-level indicators are used as proxies.

### 2.2. Collection of Evidence-Based Statistics

This step processed the collection of data and statistics, both from the related agencies involved within the study areas (e.g., [19,20,21,22,23]), and accumulated literature available in each country in their own national languages, as well as from peer-reviewed international studies that assessed the extent to which extreme events are found and the sample of countries investigated. Integrated literature reviews to provide valuable input for the cross-comparison assessment and analysis of examples were executed as they present the results of studies on emerging issues as well as recommendations for future research and fields of action from multiple perspectives [24,25].

### 2.3. Comparative Analysis of Climate Risks and Extreme Events, and Health

A comparative analysis to understand the climate-setting stressors, implications and impacts on the sample, and compilation of a list of climate risks, lessons learned and likely recommendations for the future in the sample of countries investigated was performed.

This step analysed the data on overall socioeconomic issues, vulnerability, climate risk and adaptive capacity status of the sample, and focused on climate-setting stressors, along with the implications and impacts of a list of climate risks. In respect of the sensitivity (i.e., the extent to which a country is dependent upon a sector negatively affected by climate hazards, or the proportion of the population particularly susceptible to climate-change hazards), and readiness measures (i.e., a country’s ability to leverage investments and convert them to adaptation actions by considering economic, governance and social readiness), it can be seen that all the surveyed examples show some degree of vulnerability to climate change and particularly to extreme events, which are likely to negatively influence human health, well-being and life-supporting sectors.

The compilation of sensitivity issues followed the guidelines identified by the Global Climate-Risk Index (CRI) [26], the Notre Dame University Gain Index (ND-Gain) [27] and the United Nations Human Development Index (HDI) [28]) from the sample of countries. 

Notably, the HDI integrates three socioeconomic and human development indicators: (i) the per capita parity purchase power (PPP) gross domestic product (GDP); (ii) education; and (iii) life expectancy as an indicator of health status [28], which have been successfully used for cross-comparison studies of adaptive capacity and development [3,29].

The Global CRI analyses to what extent countries were affected by the impacts of weather-related events from 1996–2015 [26]. 

The ND-Gain Index measures vulnerability (exposure, sensitivity, and adaptive capacity) and readiness (a country’s ability to leverage investments and convert them into adaptation actions) when it comes to climate change and climate-related impacts. The analysed life-supporting sectors included herein are health, food, water and human habitats. The health score captures a country’s public health vulnerability to climate change, in terms of the spread of communicable diseases and provision of health services [27].

The present study consists of a comparative analysis by looking at the links between climate risks (climate changes and variations, and extreme events) and health, with a diverse combination of industrialised, advanced transitional, developing and least-developed (LDC) nations, in order to gain an overview of the diverse impacts on the overall vulnerability, climate risk and adaptive capacity status at different geographical scales (countries, regions and cities), as shown in Figure 1.

## 3. Surveyed Samples

The results from the eight surveyed examples are presented in three sub-sections and four tables as follows:

### 3.1. Climate-Risks and Extreme Events in the Surveyed Examples

Table 1 presents both the CREE and the prioritised impacts in the surveyed examples.

### 3.2. Comparative Socioeconomic, Vulnerability, Climate Risks and Adaptive Capacity Status of the Studied Countries

Table 2 presents the comparative socioeconomic, life-supporting vulnerability sectors (health, food, water, and human habitat), climate-risk, and adaptive capacity status of the studied examples at the country level. These can be traced back to the existence—or lack of—sound climate policies, which are especially needed in developing countries. Based on the data gathered, the most relevant features presented in Table 2 are as follows:

(i) Australia shows low to moderate climate risks, very low water vulnerability and sensitivity, and very high readiness to adapt. It is the best placed of the studied countries in all indicators except food (50th) and readiness.
(ii) Austria has shown a low degree of vulnerability, particularly in health, but high water vulnerability, moderate sensitivity, and very high readiness.
(iii) Bolivia shows a high vulnerability and sensitivity, low readiness but moderate impacts.
(iv) Cameroon shows a high to very high degree of vulnerability and risks, a very low readiness to adapt, and low sensitivity to climate variations.
(v) Ethiopia shows a high to very high degree of vulnerability, particularly in the health sector, moderate risks, moderate sensitivity to climate variations, and low readiness to adapt.
(vi) Malaysia shows good development indicators, a moderate vulnerability in health, water and human habitat, and a high vulnerability in the food sector.
(vii) Tanzania shows very poor development indicators, high and very high vulnerabilities, high sensitivity to climate variations and low readiness to adapt.
(viii) Uruguay shows good development indicators, very low vulnerabilities in water and food, low in health, and high in habitat, high sensitivity to climate variations, and a good degree of readiness to adapt.

Despite large socioeconomic, vulnerability and adaptive capacity status, and climate-risk differences, ranging from a LDC (Ethiopia) to developed countries (Australia and Austria), all eight studied countries show some degree of vulnerability and are directly and indirectly impacted by climate-related hazards. Indirect impacts often are much more severe than direct ones, e.g., food shortages because of droughts [26] which are not adequately featured by the CRI [30].

Because of a low level of development and adaptive capacity, the implications and impacts of climate change and extreme events undermine further socioeconomic and human development (e.g., Ethiopia, Cameroon, Tanzania), affect the eldest, poorest and/or women (e.g., Ethiopia, Tanzania), but also socioeconomically vulnerable groups like immigrants in Austria and aborigines in Australia.

Some countries are vulnerable to mainly one cause and, therefore, are highly sensitive (e.g., Austria for extreme heat and Uruguay for flooding threats to the human habitat). Other countries suffer strong climate distress but are moderately impacted (e.g., drought and limited availability of water in southern Australia). Several surveyed examples show successful early-warning systems (EWS) or are developing them (e.g., La Paz for extreme rainfall and landslide events and Dar-es-Salaam for sea-flooding respectively).

### 3.3. Overview of the Surveyed Examples

In this section, eight examples describing climate-setting stressors, implications and impacts of climate risks and extreme events are presented.

#### 3.3.1. Austria

Austria is a landlocked country in Central Europe consisting of nine states, 83,879 km^2^, and 8.7 million people with the capital Vienna containing 1.8 million inhabitants. The climate of Vienna is transitional, with an average yearly temperature of 7.9 °C (in 2016), influenced oceanically from the west and continentally from the east. This is shown in smaller precipitates, longer periods of dryness and rather mild winters compared to other parts of Austria. Over the last decades, Austria has experienced an increase in the average air temperature [31] and current projections suggest that this trend will continue in the future. Until 2040 an annual temperature rise by about 1.6 °C is estimated [32]. Therefore, the direct health effects of climate change are the most pressing areas of action within the framework of the national adaptation strategy [33]. These effects include the acute impact of extreme temperatures, especially heat waves in urban areas, and heavy rainfall with flooding, mudflows and landslides. Particularly older adults (>65 years) with a low socioeconomic status and poor health conditions, who tend to be socially isolated, are most at risk during extreme heat events [34]. Among the elderly urban population, persons with Turkish migrant background are considered vulnerable to an even higher degree due to the intersection of several risk factors: social status (poverty, manual labour), residential area (densely populated, disadvantaged urban areas, heat islands), and health condition (chronic diseases) [35].

#### 3.3.2. Ethiopia

Ethiopia has a population of 79 million people and a surface of 1.2 million km^2^. Broadly, the Ethiopian topography is divided into peripheral lowlands and central highlands. The country has 18 agro-ecological zones (Ministry of Agriculture) [36]. The mean annual temperature of the highland and lowland is 10 °C to 30 °C, respectively. Annual rainfall ranges from 3600 mm to 400 mm. The country is renowned for its rich water resources and is known as the water tower of north-east Africa. The Ethiopian population increased annually at the rate of 3.4% from 1984–1994, 2.9% from 1994–2007 and, currently, is growing at a rate of 2.77% [37]. About 85% of the population lives in rural areas [38,39] and mixed farming and pastoralism are the mainstays of livelihoods. The agricultural and health sectors are frequently affected by climate extremes and suffer from technological limitations [39,40,41]. Ever-rising temperatures, erratic rainfall distribution, recurrent droughts, and floods are worsening access to and the quality of water, food, food insecurity and famine. The country is highly vulnerable to climate-change sensitive diseases (e.g., air pollution-related respiratory diseases, meningitis, vector-borne and water-borne diseases) [38,39,40,41,42,43].

#### 3.3.3. Malaysia

Malaysia has a population of 31.5 million people and a surface of 330,803 km^2^. It is a South-east Asian tropical country with an average yearly temperature of 27 °C, characterised by the north-east and south-west monsoon seasons. The Malaysian weather pattern is associated with El Niño and global climate change [44,45]. Most drought episodes in Malaysia are associated with El Niño, which sometimes results in haze (bushfire in Indonesia). However, the El Niño-Southern Oscillation (ENSO) is also responsible for the severity of impacts and the Ocean Dipole (IOD) could enhance the severity of a drought episode if it co-occurs with an El Niño episode (e.g., in the case of the extreme events in 1997–1998, 2000–2001, 2003–2005, 2007–2008 and 2014–2015), leading to longer dry spells. There are six climate-sensitive waterborne-, vector borne-, and heat-related diseases linked to climate change and extreme event conditions. The delayed effects of haze were found in a respiratory mortality incremental of 41.4% and 66%, respectively, of adult females aged 15–59 years [46].

The annual economic loss from the haze and health impacts is estimated to be on average US$ 0.12 million annually [47,48]. Approximately 17.6 million people are projected to be at risk and 5900 premature deaths may be attributed to climate change and extreme event-related effects by 2030 [49].

#### 3.3.4. Uruguay

Uruguay has a population of 3.4 million people and a surface of 179,000 km^2^. Average yearly temperature is 15–16 °C and precipitation is 1380 mm/year, having varied from 823 mm to 2060 mm during strong La Niña/El Niño years, respectively, from 1961–2014 [50]. The country is bordered to the west by the flood-prone Uruguay River [51,52].

The climate vulnerability of the health sector is related to extreme events and vector-borne infectious diseases (VBID). Uruguay River floods occur frequently (e.g., 1997, 2007, 2014, 2015 and 2017), many of them related to El Niño events [50]. The number of evacuated and displaced people during floods varies from 1000 to ≥12,000 but [22], because of efficient rescue and relief, the impact on the population’s health is mainly related to psychosocial stress [23,50].

Excessive rainfall favours the transmission of VBID such as Leptospirosis in flood-prone zones without good sanitation. A positive correlation was found between yearly rainfall and the number of yearly cases from 2005 to 2014 (r: 0.73; *p* < 0.02) peaking during the excessive rainfall and flooding years 2007 and 2014, and a similar but less clear relationship was observed for Hantavirus [50].

#### 3.3.5. South-Eastern Australia (SEQ)

South-East Queensland (SEQ), Australia, is a region on the east coast of Australia in the State of Queensland, consisting of 12 local government areas, 22,420 km^2^ and 3.4 million people. It has a sub-tropical climate, dry, warm winters and hot, wet summers, and the average yearly temperature is 20 °C. Rainfall and temperature are strongly influenced by decadal ENSO events. SEQ experienced both drought and flood and periods of extreme heat and prolonged periods of hot days. Current climate-change projections suggest that increased average temperatures and extremes of temperature are highly likely but impacts on rainfall are less certain [53].

Flood events in the region result in disease risks from the contamination of water, especially leptospirosis during flood events [54]. Substantial resources put into water security means drought has less direct health impacts, but there are significant mental-health problems among farmers and rural communities [55,56,57], and particularly Aboriginal communities, for whom connectedness to healthy land is essential for individual and collective health and well-being [58], and who are often more socioeconomically disadvantaged. Cases of heat-stroke deaths due to extreme heat are well-documented in the region and Australia more widely. The risk of health impacts due to heat are highest for the old and young, and the most socioeconomically disadvantaged who will have less access to air conditioning [59,60,61].

#### 3.3.6. Douala, Cameroon

Douala has a population of 2.8 million people living on a surface of 210 km^2^. It has a tropical monsoon climate, with a yearly temperature of 28 °C, characterised by heavy precipitation, especially during the rainy season from June to October. Its geographical situation in mangrove ecosystems creates sensitivity to extreme events, particularly to the rise of sea levels [62]. Floods in Douala are being associated chiefly with changing rainfall patterns, resulting from climate change [63]. During the past few decades, the city has been impacted by extreme events such as droughts, floods, heatwaves and storms, causing loss of life and materials. Due to poor hygiene and sanitation around toilet facilities, floods contaminate drinking water, resulting in the increase in the incidence of cholera and various waterborne diseases [64] as well as in the mosquito population [65]. An increase of temperatures and heat waves has led to an increase in the rate of air pollution and respiratory infections [64]. Headaches, fatigue, and the feeling of being very hot were associated with high indoor air temperature among schoolchildren in Douala [65]. The same result was found by assessing the health-related impacts of the urban heat island in Douala Metropolis [66].

#### 3.3.7. La Paz, Bolivia

La Paz is situated at 3600 m above sea level in the Bolivian highland, with a population of 1.5 million people. Average yearly temperature and rainfall are 10 °C and 571 mm/year, respectively. About 63% of the urban area consists of steep, unstable terrain, and 37% of southerly flood-prone neighbourhoods are gently sloping. Health vulnerability is high due to rapid population growth, informal settlements on unstable slopes, poor governance, lack of infrastructure, and health inequity [67,68].

Two extreme rainfalls have occurred: (i) February 2002: a heavy rainfall and hailstorm developed rapidly over the central and northern areas with a death toll of 74 people and 120 injured (traumatisms, hypothermia, depression, stress, anxiety and respiratory and cardiovascular illness). An early-warning system (EWS) and a risk map were developed to plan protective measures and land-use planning [69]; (ii) February 2011 (the rainiest February ever recorded): a heavy rainfall linked to a moderate La Niña event and mega-landslide affected 26% of the city (“Callapa region”), 6000 people, and 450 houses, 163 of which were buried. This mega-landslide developed slowly and enabled timely action and EWS activation; there were no deaths or serious health scenarios but cases of dehydration, with greater prevalence among children, diarrhoea in children under two, myositis, depression, stress, anxiety, acute respiratory infections, minor injuries, and ectoparasites among the evacuated people [69]. 

#### 3.3.8. Dar-es-Salaam, Tanzania

Dar-es-Salaam is a coastal city with 4.3 million people [70], an average yearly temperature of 26 °C; yearly rainfall is over 1000 mm/year, with two main rainy seasons: (i) the long rains (March–May), and (ii) the short rains (October–December) [71]. The low elevation (<10 m) coastal zones (LECZ) are threatened by extreme rainfall and floods associated primarily with ENSO events which impact infrastructure, the built environment and the economy [72], and sea-level rise [73] which impacts health. 

Unplanned urbanization has led to flood risk in many informal settlements where malaria is endemic, particularly during the rainy seasons. Between 2010 and 2014, severe floods affected over 50,000 people [73] and most of the reported diseases associated with floods were water-borne in nature [74]. 

About 140,000 people in Dar-es-Salaam live in LECZ, and over 30,000 are at risk [70]. Thus, the improvement of El Niño forecasting and warning systems could play a role in reducing damage in the future. The Tanzanian Meteorological Agency (TMA) provides both near-term (24 h) and seasonal forecasts, and warnings of flood events [75]. Prolonged droughts have severe socioeconomic and health implications, and the prevalence of food and waterborne diseases (WBID) such as diarrhoea, cholera, hepatitis A, and typhoid fever, as well as vector-borne diseases (VBID), mainly malaria, dengue fever and schistosomiasis, were widespread in Dar es Salaam [76]. 

#### 3.3.9. Summary of the Surveyed Examples

The analyses in the results section illustrate the fact that climatic patterns, sociodemographic and socioeconomic factors, variables, contexts and determinants are very heterogeneous on global, regional and national scales, and thus determine different options for adaptive strategies. Table 3 presents climate-related overall impacts, critical sector(s), adaptive capacity, and readiness to overcome related issues based on the information of Table 2 and the surveyed examples. 

Table 4 summarises climate-related overall impacts on human well-being, health safety, and environmental health.

## 4. Discussion

In this section due attention to three main items will be provided, drawing from the data presented in the results section, and addressing some key questions that have arisen from the study.

### 4.1. Policy Implications

First and foremost, this paper highlights how the degree of exposure to climate-risks and extreme events can be exacerbated by the lack of climate policies, especially those which fail to take into account the negative impacts of extreme events. 

Interestingly, people who have recently experienced severe weather events such as floods, storms and drought are more likely to support policies to adapt to the effects of climate change. Some authors argue about the existence of a link between extreme weather exposure and support for climate-change adaptation [77]. Given this, in order to yield the expected benefits, policies should also take into account social imbalances and the increased vulnerability of the poor [57].

Furthermore, poor countries are more heavily affected by extreme weather events and future climate change than rich countries. This discrepancy is sometimes known as an adaptation deficit [78]. There is also a deficit of understanding of how climate adaptation is taking place at a national or regional level, which is particularly pronounced in developed nations which have typically been assumed to have a low vulnerability to climate change [79]. The surveyed examples suggest the existence of an adaptation deficit to the current climate extremes beyond the coping range of both natural and human systems which is not restricted only to poor countries [48].Weather and climate extremes might be the engine to better understand climate impacts and foster adaptation by means of learning to manage risks under uncertainty [9,80,81]. 

The increase of extreme events, their likely expected future increase [9], and their implications on well-being, health and sustainable development presented in this article suggest that despite their different climatic, socioeconomic and public health status, the examples studied have been severely impacted over the last few decades, and might continue to be so in the near-future. 

The current adaptation deficit should be reduced through decoupling vulnerability to extreme events from economic growth (e.g., reducing exposure; fostering EWS; improving after-event actions).

### 4.2. Main Issues Related to Climate Change and Health Arising from the Study

From the comparative analyses above, the following questions about relevant issues arise:

(i) To what extent is the existence or lack of climate policies influencing vulnerability?
(ii) How are we looking at climate-change education and literacy, climate change capabilities and infrastructures (climate-adaptive technological change), and climate change governance?
(iii) How do we learn from different training and expertise realities, allocated resources (namely for health facilities, educational and preventive intervention), and how do we integrate/manage supranational, regional, national and local regulatory mechanisms for land use, indigenous and traditional peoples (traditional knowledge and climate change), urban planning, coastal-zone integrated management, population growth, displacements and the interactions between them?
(iv) What about climate governance? The geographies (and societies) analysed showed different vulnerabilities and response to climate change. There are geographic and socioeconomic differences in incidence, vulnerability, climate risk and adaptive capacity which arise due to different socioeconomic and human development, environmental exposure and resilience (from human systems and ecosystems—processes, services and productive capacity of the environment, settlements, infrastructure and services). Global issues are increasingly characterized by interconnectedness and complexity (e.g., forced migration and internal displacement of people, and emerging infectious diseases [82,83].

### 4.3. How to Tackle These Questions to Innovate Adaptation Policies and Strategies?

Deeper scientific integration is a prerequisite for tackling these questions. The health impacts may be controlled through adopting proactive measures and, as stated by [84]:

‘*Strengthening capacity for surveillance of diseases of relevance to local populations can provide a mechanism for building the cross-cutting and flexible capacities needed to tackle both the burden of existing diseases and emerging infectious disease threats*’.

Together with the establishment of local early-warning systems for the health effects of predicted climate change [83], this should improve how we deal with this global challenge.

Addressing climate change could be the greatest global health opportunity of the 21st century [85], since the impacts of climate change on health are very sensitive to both climate-change policy and climate and environment-related health measures.

Apart from sound policy-making, more governance and consensual governance choices are urgently needed. Innovative solutions and opportunities (e.g., climate change and governance) can be found in the United Nations Sustainable Development Goals (UN SDGs) that contain a set of 17 measures to foster sustainable development across many areas. This offers a good opportunity to reinvigorate sustainable development research for two main reasons [86]. First, it comprises many areas of sustainable development (SD) research, which have become mainstream thanks to the UN SDGs. Second, the fact that the UN and its member countries have committed to attaining the SDGs by 2030 has added a sense of urgency to the need to perform quality research on SD, on the one hand, and reiterates the need to use the results of this research on the other. An explicit and deliberate dialogue between scientists, communities, practitioners and decision-makers should lead us to ‘getting people on-side’, including local communities [87], in climate-change mitigation measures and adaptation strategies in a given context and learning from others’ experiences and best practices.

## 5. Conclusions

The connections between climate change and health exist but need to be further studied. Extreme events are known to pose an additional burden to the health systems of developing countries, which are already under severe pressure. This paper has used a variety of concrete examples to outline that there is a pressing need to address the current and future health impacts of extreme events and to consider them in future mitigation and adaptation strategies. 

However in order to yield the expected benefits, addressing the health impacts of extreme events requires a number of measures. Firstly, there is the need for sound climate policies which afford extreme events the attention they deserve. This means that due consideration to health elements should be part of climate-change mitigation strategies, on the one hand, and also that an emphasis on health aspects be part of current and future adaptation strategies. 

In addition, an integrated effort which mobilizes resources for research and for practical works at the local level is necessary. Moreover, handling extreme events requires an emphasis on prevention, since handling their consequences is not only costly but also generates additional pressures on the health of individuals, especially in developing countries where the financial resources to address these consequences are seldom available. This, again, emphasises how strategically relevant it is to link health issues with mitigation and adaptation programmes related to extreme events.

Furthermore, as this paper has highlighted, the occurrence of extreme events also has implications for the adaptive capacity of communities. This is so since chronic vulnerability makes adaptation efforts very difficult to implement. Therefore, consideration of health issues should play a stronger role in policy-making on the one hand, and on attempts to reduce the impacts of climate change at the community level on the other. Bearing in mind that the vulnerability of human and natural systems makes them more likely to suffer the impact of extreme events, there is also a need for some concrete measures such as enhanced early-warning systems for coastal areas and mountains or river floods, and the identification of particularly vulnerable social groups which deserve special attention.

Indeed, a key feature of handling the health impacts of extreme events is the reduction of the exposure of the various groups. The 2012 Intergovernmental Panel on Climate Change (IPCC) report (AR-4) has shown that settlement patterns, urbanization, and changes in socioeconomic conditions have all influenced observed trends in exposure and vulnerability to climate extremes, affording to this a high degree of confidence. Therefore, decision-makers and planners should know what to do: put the necessary mechanisms in place and take the action needed to reduce exposure, and hence minimise the vulnerability of the weakest social groups to extreme events and, inter alia, to the health problems associated with them.

The evidence gathered from the paper also suggest that, apart from the use of sound policies, strategies and processes for adapting to the impacts of climate risks and extreme events, more use should be made of regional, national and local best practices for making innovative and meaningful use of their results. Also, lessons learned from climate science and evidence provided by climate-change projections (e.g., increases in the occurrence and severity of CREE) and the described relations between the projected impacts of climate change and extreme events and socio-environmental vulnerability and disaster risk reduction (from climate science to action), should be better integrated in the definition of innovative and meaningful adaptation strategies (so as to meaningfully contribute to the debate on climate change, variability, and extreme events, and innovation for climate adaptation).

The study is limited in the sense that it includes a selection of sites. Despite this fact, the study covers a wide range of socioeconomic, climatic and geographical determinants of human well-being, health vulnerability and risks. Therefore, even though these conclusions are site-specific, the nature of the findings means that they could be useful to other sites suffering from the same problems. 

## Figures and Tables

**Table 1:** Summary of climate risks (due to climate change, variations, and extreme event stressors) in the surveyed places (source: Authors, based upon cited literature in Section 3.3). NAR: climate stressors not applicable or relevant; NAH: relevant CR whose impacts are not addressed herein; ↑: major climate risks with impacts addressed herein; ↑↑: extreme CR addressed in this study at each example in Section 3.3.

Examples/Climate Risks	Austria	Ethiopia	Malaysia	Uruguay	SEQ	Dar-es-Salaam	Douala	La Paz
Rising temperature	↑↑	↑	↑↑	NAH	↑↑	↑	↑	↑
Heatwaves	↑↑	↑	↑↑	NAH	↑↑	↑	NAH	NAH
Drought	X	↑↑	X	NAH	↑↑	↑	NAH	NAH
River flooding	↑	↑↑	↑↑	↑↑	↑↑	↑↑	↑↑	NAH
Heavy rainfall	NAH	↑↑	↑↑	↑↑	↑	↑↑	↑↑	↑↑
Erratic rainfall	NAH	↑↑	↑↑	↑	NAH	↑	NAH	NAH
Mud/landslides	↑	NAH	NAH	NAR	NAH	NAH	NAH	↑↑
El Niño–Southern Oscillation (ENSO)	NAR	NAR	↑↑	↑↑	↑↑	↑↑	↑↑	↑↑
Sea-level rise	NAR	NAR	NAH	NAH	NAH	↑↑	↑	NAR
Sea flooding	NAR	NAR	NAH	NAH	NAH	↑↑	↑↑	NAR
Monsoon	NAR	NAR	↑↑	NAR	NAR	NAR	NAR	NAR

**Table 2:** Comparative country-level socioeconomic, vulnerability, climate-risk and adaptive capacity status of the surveyed examples. Climate Risk Index (CRI) 2017: average 1996–2015. CRI score, fatalities/100,000 inhabitants, losses per unit GDP. Vulnerability (health, water, food, human habitat sectors, sensitivity to climate-related hazards), and readiness (Read): The lower the values, the lower the risks and impacts.

Countries	Human Development	Climate Risk Index [26] CRI Score	ND-Gain Index to Cope with Climate Change and Climate-Related Hazards [27]					
	Human Development Index (HDI), Class, and World Rank [28]		Vulnerability, Sensitivity (Sen) and Readiness (Read). Score and World Rank The Lowest (1st)–The Highest (192nd)					
			Vulnerability Life-Supporting Sectors				Sen	Read
	HDI Rank	Score, World Rank	Health	Water	Food	Human Habitat		
Australia	0.939, Very High, 2nd	52, 34th	0.02, 1st	0.20, 15th	0.39, 50th	0.31, 32nd	0.24, 22nd	0.75, 16th
Austria	0.893,Very High, 24th	61, 50th	0.02, 3rd	0.49, 111st	0.32, 32nd	0.32, 34th	0.34, 53rd	0.79, 8th
Bolivia	0.674, Medium, 118th	52, 37th	0.42, 132nd	0.51, 126th	0.45, 72nd	0.57, 147th	0.44, 97th	0.34, 144th
Cameroon	0.518, Low, 153rd	134, 146th	0.48, 139th	0.44, 83rd	0.66, 143rd	0.67, 176th	0.30, 34th	0.31, 160th
Ethiopia	0.448, Low, 174th	70, 66th	0.71, 188th	0.47, 97th	0.69, 153rd	0.61, 159th	0.39, 76th	0.33, 146th
Malaysia	0.789, High, 62nd	94, 103rd	0.18, 67th	0.35, 42nd	0.63, 127th	0.39, 71st	0.26, 25th	0.59, 41st
Tanzania	0.531, Low, 151st	103, 116th	0.67, 175th	0.49, 112nd	0.70, 160th	0.59, 153rd	0.44, 99th	0.36, 132nd
Uruguay	0.795, High, 54th	82, 86th	0.14, 55th	0.30, 27th	0.31, 28th	0.54, 137th	0.44, 97th	0.57, 44th

**Table 3:** Summary of climate-related overall vulnerability and sensitivity (critical sectors), climate stressors, implications and health impacts, and overall adaptive capacity and specific readiness in the case studies examined. The main climate stressors are based on Table 1 and the development, vulnerability, adaptive capacity statuses are based on Table 2 and issues presented in the surveyed examples. The sensitivity is equally weighted and averaged. Vector-borne and water-borne infectious diseases: VBID and WBID, respectively.

Surveyed Examples	a. Climate-Related Vulnerability b. Sensitivity (Critical sectors), and c. Risk	Main Climate Drivers and Stressors	Implications and Impacts on Human Well-Being and Health	a. Overall Adaptive Capacity b. Specific Readiness
Austria	a. Low b. High (Water) c. Low	Rising temperature, heatwaves, flood, mud- and landslide	Heat stroke; post-traumatic stress syndrome	a. Very High b. High
Ethiopia	a. Highb. High (Health, Food, Habitat) c. Medium	Rising temperature, heatwaves, strong erratic rainfall variability, floods and drought	Food diseases; VBID; WBID; malnutrition; occasional famines	a. Very Low b. Very Low
Malaysia	a. Low b. Medium (Food) c. High	Rising temperature, monsoon, El Niño, heat waves, rainfall, floods, bushfire	Climate-sensitive, WBID, VBID, and heat-related diseases	a. High b. High
Uruguay	a. Low b. High (Habitat) c. Medium	El Niño; heavy rainfall, river flooding	Displaced people; habitat damage and loss; psycho-stress, VBID	a. High b. High
SEQ, Australia	a. Low b. High (Food) c. Low	Rising temperature, ENSO, heatwaves, drought, flood	Displaced people; habitat damage and loss; psycho-stress; VBID	a. Very High b. High
Douala, Cameroon	a. Medium b. High (Habitat) c. Very high	Heavy rainfall, river and sea-flooding, sea-level rise	Shortage of irrigation and drinking water; VBID; WBID	a. Very Low b. Low
La Paz, Bolivia	a. Medium b. High(Habitat) c. Low	ENSO; rainfall, landslide	Displaced people; injuries; habitat damage and loss; psycho-stress	a. Very low b. High
Dar-es-Salaam, Tanzania	a. High b. High (Health, Food, Water, Habitat) c. High	ENSO-variability linked extreme rainfalls, river floods, droughts, sea-level rise	Climate-sensitive, VBID, WBID	a. Low b. Very Low ^a^

Note: ^a^ The current development of an early-warning system should reduce vulnerability, impacts and risks.

**Table 4:** Summary of overall impacts on well-being and environmental health.

Surveyed Examples	Data Collected Show the Following Impacts:
Austria	Mean temperature, the frequency of extreme heat events, heavy rainfalls, flooding, and landslides are increasing. Health-related risks caused by heatwaves are enhanced by several factors including social status (poverty, manual labour), residential area (densely populated, disadvantaged urban areas, heat islands) and health condition (chronic diseases) [35].
Ethiopia	Heatwaves, cardiovascular and respiratory diseases, malnutrition, and VBID are among most observed climate-sensitive diseases [42]. The situation is worst among the marginalized and poor due to very weak adaptive capacity [43].
Malaysia	West and East Malaysia experienced a significant increase of mean temperature, uneven rainfall, prolonged dry spell, El Niño and extreme events [44,45,47]. Those are causing waterborne, VBID, and heat-related diseases. The existing system is not ready yet to prevent climate change-related vulnerability [47].
Uruguay	Emergency response to climate change and variability, respectively [22,23]. Nevertheless, they still lack effective pre-event readiness [29]. VBID has increased after the El Niño events of 1997 and 2002, varying since 2005 in relation to total yearly rainfall [50].
SEQ, Australia	There are widespread infections due to flooding [54], heat-related deaths in heatwaves [59,60,61], and growing mental-health impacts due to drought [55,56,57]. The most vulnerable people are the elderly, agricultural communities and indigenous groups [56,58].
Douala, Cameroon	Facing severe health problems like heat-related illness and waterborne diseases due to the occurrence of extreme events, especially when there are few or no means of protection and adaptive actions [63,64,65,66].
La Paz, Bolivia	The municipal administration has achieved a successful adaptation over the last decade through adaptation actions: (i) early warning of heavy rainfall, (ii) risk mapping, and (iii) a rescue and relief sub-system [69].
Dar-es-Salaam, Tanzania	Communities are vulnerable to waterborne diseases caused by floods [42,43]. Transmission occurs throughout the year, with a seasonal increase in intensity that coincides with the two rainy seasons, March to May and October to December [71].
